# SARS-CoV-2 and human milk: what is the evidence?

**DOI:** 10.1101/2020.04.07.20056812

**Published:** 2020-04-11

**Authors:** Kimberly A. Lackey, Ryan M. Pace, Janet E. Williams, Lars Bode, Sharon M. Donovan, Kirsi M. Järvinen, Antti E. Seppo, Daniel J. Raiten, Courtney L. Meehan, Mark A. McGuire, Michelle K. McGuire

**Affiliations:** 1 Margaret Ritchie School of Family and Consumer Sciences, University of Idaho, Moscow, ID, USA; 2 Department of Animal and Veterinary Sciences, University of Idaho, Moscow, ID, USA; 3 Department of Pediatrics and Larsson-Rosenquist Foundation Mother-Milk-Infant Center of Research Excellence (MOMI CORE), University of California, San Diego, La Jolla, CA, USA; 4 Department of Food Science and Human Nutrition and Institute of Genomic Biology, University of Illinois, Urbana, IL USA; 5 Department of Pediatrics, Division of Allergy and Immunology, University of Rochester School of Medicine and Dentistry, Rochester, NY, USA; 6 Eunice Kennedy Shriver National Institute of Child Health and Human Development (NICHD), National Institutes of Health (NIH), Bethesda, MD, USA; 7 Department of Anthropology, Washington State University, Pullman, WA, USA

## Abstract

The novel coronavirus SARS-CoV-2 has emerged as one of the most compelling public health challenges of our time. To address the myriad issues generated by this pandemic, an interdisciplinary breadth of research, clinical, and public health communities have rapidly engaged to find answers and solutions. One area of active inquiry is understanding the mode(s) of SARS-CoV-2 transmission. While respiratory droplets are a known mechanism of transmission, other mechanisms are possible. Of particular importance to global health is the possibility of vertical transmission from infected mothers to infants through breastfeeding or consumption of human milk. However, there is limited published literature related to vertical transmission of any human coronavirus (including SARS-CoV-2) via human milk and/or breastfeeding. There is a single study providing some evidence of vertical transmission of human coronavirus 229E, a single study evaluating presence of SARS-CoV in human milk (it was negative), and no published data on MERS-CoV and human milk. There are 9 case studies of human milk tested for SARS-CoV-2; none detected the virus. Importantly, none of the published studies on coronaviruses and human milk report validation of their analytical methods for use in human milk. These reports are evaluated here, and their implications related to the possibility of vertical transmission of coronaviruses (in particular, SARS-CoV-2) during breastfeeding are discussed.

## INTRODUCTION

The global pandemic caused by the SARS-CoV-2 virus is one of the most compelling and concerning global health crises of our time. Fortunately, this pandemic has rapidly engendered a mobilization of the full range of expertise represented by research, clinical, and public health experts. While our understanding of the biology, clinical implications, and strategies for mitigation continues to evolve, one issue that has received limited attention is the implication of this pandemic for infant feeding practices. This lack of attention has resulted in mixed messages regarding guidance about optimal infant feeding practices^1,2^ and a consequent lack of confidence about the best approaches to infant feeding in the face of this growing pandemic.

Several issues related to this topic demand immediate attention, the first and foremost of these being whether or not the virus is present in human milk. Of particular interest in this context are 1) the potential role that breastfeeding could play in vertical transmission of SARS-CoV-2 from women to infants via human milk; and 2) the potential protective effects of targeted antibodies and other immunoprotective components in human milk against COVID-19. The goal of this review is to evaluate the published evidence regarding the presence of this and other human coronaviruses in human milk.

## METHODS

### Search strategy and selection criteria

We used both Google Scholar and PubMed to identify relevant literature published as of April 4, 2020. Because some of the reports relating to SARS-CoV-2 have not yet been published in refereed journals (i.e., gray literature), we also used a general Google search and a search of preprint servers bioRxiv and medRxiv. The list of search terms used can be found in Table 1. Any research in which human milk was collected and tested for a human coronavirus was included in this review.

## RESULTS

### Overview of vertical transmission of viruses (other than coronaviruses) via human milk

It is well established that viral transmission through human milk can occur.^3,4^ Notable examples include human immunodeficiency virus (HIV),^5,6^ cytomegalovirus (CMV),^7^ and human T-cell lymphotropic virus type 1 (HTLV-1).^8^ Perhaps the most prominent example of mother-to-child viral transmission via breastfeeding is HIV, where higher maternal milk and serum viral loads are associated with an increased risk of transmission.^9–11^ The risk of postnatal infection for breastfed infants of HIV+ mothers is ~10–20% over the first 2 years of life.^12,13^ However, compared to mixed feeding, exclusive breastfeeding is associated with lower risk of transmission of HIV infection to infants.^14,15^ In many high-income nations, breastfeeding is contraindicated in the case of maternal HIV infection.^16,17^ However, in low-and-middle-income nations, infant mortality from malnutrition and infectious disease may outweigh the risk of acquiring HIV.^16,18^

With respect to CMV, it is estimated that ~60–70% of breastfed infants of CMV-seropositive infants become infected with CMV.^19,20^ The risk of CMV infection in neonates is highest in preterm or very low birthweight (<1500 g) infants;^21,22^ in a small percentage of infections, infants develop a severe complication known as CMV sepsis-like syndrome, which can be fatal.^23^ Nonetheless, breastfeeding is not contraindicated in CMV-seropositive women with healthy, term infants.^16,24,25^

For HTLV-1, breastfeeding is considered the major route of infection for infants.^26^ HTLV-1 infection is lifelong, and while most infected individuals remain asymptomatic, ~10% develop severe disease, including adult T-cell leukemia, a highly aggressive and usually fatal malignancy.^27^ Some organizations and agencies list maternal HTLV-1 as a contraindication for breastfeeding,^16,28^ while others do not.^25^

### Human coronaviruses and their vertical transmission

Human coronaviruses are enveloped, positive-sense, single-stranded RNA viruses first described in 1965.^29^ There are 7 identified strains known to infect humans. Four of the strains (alphacoronaviruses 229E, NL63, and OC43; betacoronavirus HKU1) are ubiquitous in humans and cause the common cold. There is limited evidence that one of these (229E) may be vertically transmitted from mothers to infants, although the mechanism remains unclear.^30^ The presence of 229E in neonatal gastric samples suggests that one possible mechanism for infection is through human milk, although this study^30^ did not specifically evaluate the presence of 229E in human milk.

### SARS-CoV

In addition to those that cause the common cold, more virulent strains of human coronaviruses have emerged zoonotically since the early 2000s, the first being SARS-CoV in 2003. The first reports of SARS-CoV were from China, although the disease (severe acute respiratory syndrome, SARS) quickly spread globally. SARS is clinically manifested by fever, dry cough, headache, muscle aches, and difficulty breathing. No treatment exists except supportive care, but there have been no reports of SARS-CoV transmission since 2004. The case fatality rate of SARS is estimated at 10%.^31^

Currently, there is one report in which human milk was tested for SARS-CoV,^32^ and two reports of human milk being tested for SARS-CoV antibodies.^32,33^ The former is a case study of a woman infected during the second trimester of pregnancy (19 wk). A single milk sample was collected 131 days after the onset of symptoms, but no additional detail on the collection methodologies was provided. Milk was submitted to the US Centers for Disease Control and Prevention (CDC), where it was analyzed using reverse transcriptase polymerase chain reaction (RT-PCR) for viral nucleic acids, and enzyme immunoassay and indirect immunofluorescence to evaluate antibody presence. No additional details on analytical methods were provided. While no viral RNA was detected, antibodies to SARS-CoV were identified in the milk. The infant in this study was never tested for SARS-CoV infection. The latter study was a case report of a 38-yr-old infected in the first trimester of pregnancy (7 wk). She recovered fully and delivered a healthy male infant at 36 wk of gestation. Milk samples were collected at 12 and 30 d postpartum and tested for SARS-CoV antibodies; all were negative. No details on the collection or analysis of the milk were provided. The infant in this study tested negative for SARS-CoV. In both of these studies, it is possible that the women had stopped shedding the virus before the milk samples were collected, as SARS-CoV shedding in other biological samples typically peaks 12–14 d after the onset of disease.^34^ There are no documented cases of vertical transmission of SARS-CoV between mothers and infants.^35^

### MERS-CoV

A related virus, MERS-CoV, emerged in Saudi Arabia in 2012. The disease caused by MERS-CoV, Middle Eastern respiratory syndrome (MERS), is characterized by severe respiratory illness with symptoms of fever, cough, and shortness of breath. Like SARS-CoV, MERS-CoV is a betacoronavirus. The case fatality rate of MERS is 34%.^31^ There are no reports, to our knowledge, of the presence or absence of MERS-CoV in human milk. However, there are reports of the presence of MERS-CoV in the milk of dromedary camels (*Camelus dromedaries*).^36–38^ There is one report of a human likely infected through the consumption of raw (unpasteurized) camel milk.^39^ In camel milk samples spiked with MERS-CoV, viable virus could still be recovered after 48 hr.^40^ These two observations have resulted in recommendations against consuming raw, unpasteurized camel milk. ^41^ It is unclear if there is vertical transmission of MERS-CoV between camelid cows and their calves, and whether infection occurs as a direct result of lactation/nursing in this species. There are no data on vertical transmission of MERS-CoV between women and their infants.^35,42^

### SARS-CoV-2

The novel coronavirus, SARS-CoV-2, was named after SARS-CoV due to its shared sequence homology (77.9%)^43^ and similar clinical characteristics. The first reported cases of SARS-CoV-2 infection emerged in late 2019 in China. While the case fatality rate for COVID-19 (the disease caused by the SARS-CoV-2 virus) is much lower than those of SARS and MERS at ~2%,^31^ the spread of this pathogen has been much more rapid and extensive.

At the time of writing, there were 9 studies that reported direct testing of milk produced by women who were infected with SARS-CoV-2^44–49^ or by women whose infants were infected.^50–52^ In total, 23 milk samples produced by 16 women have been tested; all were negative for the presence of the virus. A description of the relevant characteristics for the women and infants in these studies can be found in Table 2. Six of the nine studies analyzed milk samples collected at birth or shortly thereafter, reporting only findings in colostrum or colostrum and transitional milk. Those same six studies reported on the milk produced by women who were infected during the third trimester of pregnancy, while the other three report findings from milk produced by mothers of infants infected at 1.5, 3, and 6 mo of age.^50–52^ For the infants born to women infected during pregnancy, most were immediately separated from their mothers post-delivery and were not breastfed for the duration of the period observed in their respective reports. Twelve of the 16 infants described in these reports were born via cesarean section, and only one was specified as a vaginal birth. Repeated milk samples were analyzed for 4 of the women, collected up to 16 days apart. All the studies were conducted in China^44–49,51,52^ or Singapore.^50^

Wang and colleagues^44^ described a healthy, 34-yr-old woman who acquired the infection in week 40 of pregnancy. She gave birth to a male infant via cesarean section. The infant and his mother both tested positive for SARS-CoV-2 using pharyngeal swabs within 36 hr of the delivery. The infant was separated from his mother at delivery and fed formula for the duration of the period described in the study. The mother’s milk was collected at 36 hr postpartum; it tested negative for SARS-CoV-2 via RT-PCR. No description of the collection or testing methods was provided. The authors stated that they recommended that the mother not breastfeed, but instead pump milk to avoid mastitis.

In another case series from China, Fan and colleagues^45^ reported on two women who became infected during the third trimester of pregnancy. Patient 1 was 34 yr old and in week 37 of gestation at the time of diagnosis. She delivered a female infant via cesarean section 6 d after testing positive for SARS-CoV-2 via nasopharyngeal swab. The infant was separated from the mother immediately after delivery, and serial tests of the infant’s nasopharyngeal swabs were negative. A milk sample was collected within 24 hr of delivery and 16 d later; both were negative for SARS-CoV-2. Patient 2 was 29 yr old and in week 36 of gestation at the time of diagnosis. Her infant was delivered 5 d after she was diagnosed via RT-PCR analysis of a nasopharyngeal swab. A single milk sample was collected within 24 hr of delivery; it tested negative for SARS-CoV-2. The authors of this report did not specify how the sample was collected, other than “breastmilk was obtained after the first lactation.”

Chen and colleague^46^ have provided the most extensive report to date, including data on milk produced by 6 women infected during pregnancy. The women were 26–34 yr-of-age and between 36 wk 2 d and 39 wk 4 d of gestation at diagnosis. The authors did not provide details on the methods used for milk collection, other than “breastmilk samples from patients with COVID-19 pneumonia were collected after their first lactation” and that milk was collected following World Health Organization (WHO) guidelines, but they did not provide a citation for this collection method. All milk tested negative for the virus, but no information was provided on the methods used for analysis.

In a report by Liu et al.,^47^ milk produced by two women was tested. One woman was 34 yr old and at 40 wk gestation tested positive via oropharyngeal swab. Milk was collected and tested from this women at d 1, 2, and 12 postpartum; all samples were negative. Her male infant was delivered via cesarean and tested for SARS-CoV-2 via oropharyngeal swab when he was 1 and 7 days old; both swabs were negative. The other woman was a 30 yr old and delivered an infant vaginally after testing positive for SARS-CoV-2. Her infant tested negative at birth using an oropharyngeal swab; milk was collected on d 2 postpartum, it was also negative. Details were provided for neither the methods of collection nor analysis.

In a research letter by Li and colleagues,^48^ information was provided related to a 30-yr-old woman at 35 wk gestation who was positive for SARS-CoV-2 and delivered a male infant via emergency cesarean section. The infant was tested immediately upon delivery via oropharyngeal swab, which was negative. After delivery, the infant was kept in isolation away from his mother. Milk was collected immediately after delivery and on d 2 and 3 postpartum; all samples were negative. Again, no information on the collection or testing methods for the milk sample is available in this report.

In another research letter, Dong and colleagues^49^ report on a 29-yr-old woman at 34 wk of gestation who was diagnosed with COVID-19 via nasopharyngeal swab. Nearly a month later, the woman delivered a female infant via cesarean section. The infant was immediately separated from the mother with no contact. The infant consistently tested negative for SARS-CoV-2 via nasopharyngeal swab over the first 12 d of life. However, a blood sample at 2 hr of age was positive for IgG and IgM antibodies to SARS-CoV-2. A milk sample was collected from the mother at d 6 postpartum; it tested negative. No information on the collection or testing methods for the milk sample is included in this report.

While the previous reports focused on infected women, there are also three case studies focused on infected infants. In these studies, milk produced by the infants’ mothers was tested for SARS-CoV-2. The youngest of these infants was reported by Cui and colleagues.^51^ After being exposed to infected family members, the 55-d-old female was admitted to the hospital with symptoms of COVID-19 and diagnosed based on clinical data and exposure history. She was “mixed fed.” Her mother’s milk was collected on the first 3 consecutive days of her hospitalization; all milk samples tested negative for SARS-CoV-2. No information on the collection or testing methods for the milk sample is included in this report. Yuehua and colleagues^52^ reported on a 3-mo-old, breastfed female who was hospitalized and tested via throat swab for SARS-CoV-2; the swab was positive. A single milk sample was collected from the infant’s mother; it tested negative. The authors provided no information on the collection or testing methods for the milk. Importantly, this infant developed symptoms of COVID-19 7 d before her parents did. As such, one possibility is that she was infected first and passed the infection to them. Another case report on a mature milk sample comes from Singapore.^50^ This report is particularly interesting as the infant had no symptoms but was hospitalized and tested because his caregivers were all hospitalized with COVID-19 and there was no one to care for him. The infant was 6 mo old and presumably at least partially human milk fed as a sample of milk was successfully collected from his mother. Despite being asymptomatic, a nasopharyngeal swab taken from the infant was positive for SARS-CoV-2. The authors reported that milk produced by the mother on a single day tested negative for the virus but do not specify how many samples were taken. This report provided no data on the methods used for the collection and analysis of these sample(s).

## DISCUSSION

Despite the devastating clinical manifestations of SARS-CoV, MERS-CoV, and SARS-CoV-2, there remains much to be learned about their modes of transmission. Respiratory droplets are a documented source of the virus, but other sources such as human milk may exist. The primary purpose of this review was to examine the evidence (or lack, thereof) for the vertical transmission of SARS-CoV-2 from mother to infant via breastfeeding considering what is known about other human coronaviruses.

There are currently 9 studies available on SARS-CoV-2 and human milk, collectively encompassing at least 23 milk samples, all of which tested negative for SARS-CoV-2. There are no comparable data for MERS, and a single case report for SARS, which yielded a negative result for the presence of the virus but positive results for antibodies specific to SARS-CoV. There have been no antibody tests in milk specific to SARS-CoV-2 in any of the reports to date, although one paper reported on SARS-CoV-2 antibodies in infant serum which were likely transplacental and thus maternal in origin.^49^ This remains a critical area that must be addressed to fully understand the role, if it exists, of breastfeeding and the feeding of human milk in infant infection.

The presence of viable MERS-CoV in camel milk is suggestive of the possibility that SARS-CoV-2 could be present and viable in human milk (or that of other species; to date, the authors are unaware of any such reports). Notably, Reusken and colleagues^36^ reported that milk was not collected from camels aseptically; rather, samples were obtained according to local milking customs. As such, it is possible that the presence of MERS-CoV in camel milk could be due to contamination from the milker, the calf, or the environment, rather than milk representing an endogenous source of the virus. However, the limited data available on all three of these viruses (and human coronaviruses, in general) leave many questions unanswered with respect to the role, if any, of human milk in vertical transmission of coronaviruses.

One possible reason that the RT-PCR results for all the milk samples tested were negative is that the methods used were neither designed nor validated for human milk. Milk is a complex matrix containing substantial fat, DNases,^53^ and RNases,^54–56^ and other PCR inhibitors.^57–59^ Thus, validation of methods using human milk is needed. In addition, other than general statements about the timing of collection (e.g., “milk was collected after the first lactation”) and brief descriptions of the RT-PCR assays used for nasal and throat swabs, none of the studies to date has described the methods of collection and how the milk was handled and stored in any detail. Of note is the fact that commonly used silica column-based RNA isolation methods are designed for a limited sample volume, and as such are not suitable for more voluminous liquid samples. In addition, nothing is known about stability of SARS-CoV-2 in human milk and how quickly (or at what temperature) it must be frozen to preserve fidelity. Information on sample collection, handling, and storage is critical to evaluating whether the negative results described in these studies could be due to inadequate methods used.

Another possibility is that there is low abundance of the virus in human milk, and it has simply not been captured in the limited samples tested so far. For example, in the report on other human coronaviruses by Gagneur and colleagues,^30^ 159 maternal-infant dyads were tested (including 161 infants, two sets of twins). In this report, 229E was present in both maternal and infant samples in only 2 dyads. Additionally, in the milk of dromedary camels, MERS-CoV appears to be present at very low abundance.^36^ This suggests the possibility that very low viral load in milk might also lead to an inflation of false negatives.

From the limited data on SARS-CoV, it appears that the presence of antibodies in milk could be influenced by timing of infection, where antibodies to SARS-CoV were detected only in milk produced by a woman who acquired the infection later in pregnancy. While the methods used to test this milk were not fully described, this observation could have impacts on the clinical management of infants born to women diagnosed with COVID-19 during pregnancy and/or lactation. This observation is also supported by the findings of Dong and colleagues^49^ who reported that both IgG and IgM antibodies to SARS-CoV-2 were present in the serum of an infant at 2 hr of age, despite multiple negative RT-PCR tests of nasopharyngeal swabs over the first days of life. The presence of antibodies at such an early stage of life could indicate transfer of SARS-CoV-2-specific antibodies from mother to infant during gestation. It is noteworthy that IgM antibodies^49^ present in the serum of SARS-CoV-2 negative infants cannot cross the placental barrier.^49^ Together, these observations suggest infant infection *in utero*, but that the virus may simply be absent from the upper respiratory tract immediately after birth.

Very recent work has demonstrated that, like SARS-CoV and human coronavirus NL63,^60^ angiotensin-converting enzyme 2 (ACE2) is one of the receptors used by SARS-CoV-2 to enter host cells.^60–62^ ACE2 is expressed across many body sites and tissue types, including the oral cavity (e.g., tongue and oral mucosa) and in mammary tissue.^63^ If mammary epithelial cells express this receptor, then it follows that viable virus could exist in milk. If it does, then the introduction of virus-containing human milk could represent a mechanism of entry for SARS-CoV-2 and COVID-19 infection for infants.

Another observation worth considering is that in at least one of the reports^52^ the infant was infected and symptomatic 7–8 days prior to the infant’s parents. This suggests the possibility that a “reverse” vertical transmission from infant to mother could occur, a phenomenon which has been observed for other pathogens, such as HIV^64,65^ and Ebola virus.^66^ One possible mechanism for maternal infection in this case is through retrograde flow, where milk and saliva move back into the mammary gland from the infant’s mouth during suckling.^67^ While this mechanism is speculative, it represents a possible route whereby an infant could theoretically transfer a pathogen it has encountered in the environment to the mother. It is also possible that maternal infection could occur through other mechanisms, such as infant respiratory droplets^68^ or via fecal matter.^69^

## CONCLUSIONS

Human milk is the gold standard for infant feeding. However, confidence with regard to its safety and best practices around breastfeeding during maternal COVID-19 infection has been compromised by the lack of evidence as to whether SARS-COV-2 can be vertically transmitted in milk and/or during breastfeeding. As such, there exists an immediate need to rapidly generate rigorous evidence for the role (if any) of human milk and breastfeeding in vertical transmission of COVID-19 from mothers to infants. To accomplish this, validation of analytical methods for the human milk matrix, viability testing, and evaluation of other immune components in milk will all be critical to this effort, especially given the known protective effects of breastfeeding in other infant respiratory infections.^70,71^ Substantial interdisciplinary research on this topic is requred and should be performed rigorously and rapidly to best inform policies regarding early feeding choices and clinical management of breastfeeding mothers infected with SARS-CoV-2 and their infants.

## Figures and Tables

**Table 1.** Search terms used in combination to identify existing literature reporting the possibility of vertical transmission of coronaviruses from mother to infant during breastfeeding. Google Scholar and PubMed were searched to identify literature published as of April 4, 2020. Preprint servers bioRxiv and medRxiv were also searched to identify preliminary reports that have not undergone the traditional peer-review process.

General Breastfeeding Terms	SARS-CoV-2 and General Coronavirus Terms	SARS-CoV Terms	MERS-CoV Terms
*milk*	SARS-CoV-2	SARS-CoV	MERS-CoV
*human milk*	coronavirus	SARS	MERS
*breast*	novel coronavirus	SARS-CoV-1	
*breastfeeding*	human coronavirus		
*breastmilk*	COVID-19		
*lactation*	COVID		
*virus transmission*			
*mother-to-child*			
*child-to-mother*			
*vertical*			

**Table 2.** Characteristics of women and infants for whom human milk has been sampled and tested for SARS-CoV-2 using RT-PCR.

*Publication*	Subjects (*n*)	Location	Repeated samples	Time postpartum	Maternal age (yr)	Gestational age at time of maternal infection	RT-PCR results	Infant age at the time of infant infection	Infant sex	Delivery mode	Infant breastfed
Wang et al., 2020	1	China	no	36 hr	34	40 wk	negative	NA	male	cesarean	no
Fan et al., 2020	2	China	yes	d 1, 17	34	37 wk	negative	NA	female	cesarean	no
		China	no	d 1	29	36 wk	negative	NA	female	cesarean	no
Kam et al., 2020	1	Singapore	no	6 mo	NS	NS	negative	6 mo	male	NS	yes^1^
Cui et al., 2020	1	China	yes	55–57 d	NS	NS	negative	50 d	female	NS	yes
Chen et al., 2020	6^2^	China	no	d 1	27	38 wk, 2 d^3^	negative	NA	NS	cesarean	NS
		China	no	d 1	26	36 wk, 2 d^3^	negative	NA	NS	cesarean	NS
		China	no	d 1	26	38 wk, 1 d^3^	negative	NA	NS	cesarean	NS
		China	no	d 1	26	36 wk, 3 d^3^	negative	NA	NS	cesarean	NS
		China	no	d 1	28	38 wk^3^	negative	NA	NS	cesarean	NS
		China	no	d 1	34	39 wk, 4 d^3^	negative	NA	NS	cesarean	NS
Liu et al., 2020	2^4^	China	yes	d 2, 3, 12	34	40 wk	negative	NA	male	cesarean	no
		China	no	d 2	30	37 wk	negative	NA	unclear	vaginal	NS
Li et al., 2020	1	China	yes	d 1, 2, 3	30	35 wk	negative	NA	male	cesarean	NS
Yuehua et al., 2020	1	China	no	3 mo	NS	NS	negative	3.5 mo	female	NS	yes
Dong et al., 2020	1	China	no	6 d	29	34 wk, 2 d	negative	NA	female	cesarean	no

Abbreviations: NS, not specified; NA, not applicable

1 The infant’s breastfeeding status was not specified in the report, but it is presumed that he was at least partially breastfed as the mother was producing milk at 6 mo postpartum.

2 Study presented data from 9 women but only had data on the milk produced by 6 women.

3 Gestational age upon admission.

4 Study presented data from 3 women but only had data on the milk produced by 2 women.
